# Identifying and characterizing clinical subgroups in individuals with endometriosis

**DOI:** 10.3389/fpain.2025.1610109

**Published:** 2025-12-12

**Authors:** Sophia Åkerblom, Ingrid Peppler Jönsson, Åsa Ringqvist, Johanna Nordengren, Xiang Zhao

**Affiliations:** 1 Department of Pain Rehabilitation, Skåne University Hospital, Lund, Sweden; 2 Department of Health Sciences, Lund University, Lund, Sweden; 3 Department of Clinical Sciences, Lund University, Malmö, Sweden; 4 Department of Obstetrics and Gynecology, Skåne University Hospital, Malmö, Sweden; 5 Institute of Psychology, University of Klagenfurt, Klagenfurt am Wörthersee, Austria; 6 Research Institute of Humanities and Social Sciences, University of Sharjah, Sharjah, United Arab Emirates

**Keywords:** endometriosis, biopsychosocial model, pain intensity, pain extent, pain catastrophizing, depression, anxiety

## Abstract

**Background:**

Classification attempts and treatment strategies for endometriosis have been predominantly biomedical. Symptom profiles observed in individuals with endometriosis are multidimensional and may be more effectively captured by a biopsychosocial model.

**Methods:**

The aim of this study was to identify distinct subgroups of individuals with endometriosis based on their biopsychosocial profiles, using Latent Class Analysis. In a subsequent phase, the identified subgroups were compared in terms of sociodemographic characteristics and various indices of functioning.

**Results:**

Two distinct subgroups were identified: Class 2, representing a high biopsychosocial burden (BPS) group characterized by both significant psychological strain and severe pain characteristics, and Class 1, representing a low BPS group with low scores on these indicators. The high BPS group reported worse control/powerlessness and greater deficits in social support.

**Conclusion:**

Moving forward, clinical assessment of patients with endometriosis may benefit from integrating core principles from the biopsychosocial model. This approach can help identify individuals facing significant psychosocial challenges who may require multidisciplinary interventions alongside evidence-based biological treatments.

## Introduction

Endometriosis is an inflammatory disease in which endometrial-like tissue grows outside the uterus. It is a common gynaecological disorder with a prevalence of approximately 10% in women of reproductive age (1, 2). Pain is a predominant feature, and both its intensity and other associated symptoms often fluctuate cyclically. Research shows that a substantial subgroup of individuals with endometriosis experience chronic pain, defined as pain lasting more than three months (3, 4). In recent decades, the scientific understanding of pain in endometriosis has shifted, since studies have demonstrated a weak correlation between lesion characteristics and pain severity. As pain becomes chronic for some patients, central nervous system mechanisms, such as central sensitization, are thought to become more influential. However, many clinicians and patients still believe that endometriosis-associated pain is solely due to the lesions, and most research and reviews continue to focus on lesion characteristics, rather than the pain (3, 5).

Beyond the physical symptoms, individuals with endometriosis often experience reduced quality of life and negative impacts on important life areas, such as relationships, sexuality, fertility, work, and leisure activities (6, 7). Individuals with endometriosis have also been shown to suffer from psychiatric comorbidities, such as anxiety and depressive disorders (8, 9), and often experience heightened pain catastrophizing (10). These clinical factors likely have a significant effect on disability and quality of life (10–12). Still, classification attempts and treatment strategies for endometriosis have primarily been biomedical, including hormone therapy, surgical methods, analgesics and in some instances opioids. The latter with negative side effects for a subgroup of patients (13–17).

The variable effectiveness of current treatment interventions suggests a need for greater optimization and personalization to improve outcomes for individuals with endometriosis (18–20). In line with this, a growing body of literature highlights the multidimensional nature of symptom profiles among individuals with endometriosis (8, 9, 21). These profiles may be better understood through a biopsychosocial model, which considers biological (e.g., pain intensity, disease severity), psychological (e.g., anxiety, depression), and social factors (e.g., financial hardship) and their interactions. The biopsychosocial burden of a specific individual is a multidimensional experience, encompassing the cumulative impact of these factors (22).

Subgrouping patients can be useful to capture the core aspects of symptoms experienced by individuals with endometriosis, enable more effective and personalized interventions, and support the healthcare system in optimizing costs and resources (23). Notably, Bendifallah et al. (24) proposed a model that focuses on clinical and symptomatic indicators to predict endometriosis using machine learning techniques. However, self-perceived psychological factors, such as pain catastrophizing, were not included in the model (24). This exploratory study aims to identify distinct subgroups of individuals with endometriosis, receiving care at a tertiary clinic, based on their biopsychosocial profiles, using Latent Class Analysis (LCA). Clinical factors, including biological characteristics (pain intensity and extent) and psychological variables (depression, anxiety, and pain catastrophizing), will serve as indicator variables (12). In a subsequent phase, the identified subgroups will be compared in terms of sociodemographic characteristics and various indices of functioning.

## Methods

### Participants

This cross-sectional study included patients referred for evaluation at the Endometriosis Clinic, Skåne University Hospital, Malmö, between April 2019 and May 2024. To be eligible, participants were required to meet the following inclusion criteria: (1) Referral to the clinic during the study period; (2) A confirmed diagnosis of endometriosis, verified either by: laparoscopic surgery, or ultrasound. The clinic is a government-supported, regional tertiary care center providing services for adults with endometriosis. All participants gave informed consent, and the study was approved by the Swedish Ethical Review Authority (2019-00023).

### Measures

All measures were based on self-report.

### Indicator variables

#### Biological factors

*Pain Extent,* or the number of pain locations was measured using 36 predefined anatomical areas (18 on the right side and 18 on the left side of the body) where the patients recorded areas with pain: (1) head/face, (2) neck, (3) shoulder, (4) upper arm, (5) elbow, (6) forearm, (7) hand, (8) anterior aspect of chest, (9) lateral aspect of chest, (10) belly, (11) sexual organs, (12) upper back, (13) lower back, (14) hip/gluteal area, (15) thigh, (16) knee, (17) shank, and (18) foot. The number of pain locations reported by the respondent (range: 0–36) was summed.

*The Numerical Rating Scale (NRS)* was used to assess self-reported pain intensity. Respondents were asked to rate their pain during the past week on an 11-point scale, where 0 = no pain and 10 = worst possible pain. The NRS is widely used in pain research and is an acceptable measure of pain intensity with demonstrated sensitivity to subjective changes in pain across various contexts (25, 26). Regarding pain intensity, the following cut-off points were used: 0–3 for mild pain, 4–6 for moderate pain, 7 and above for severe pain (27).

#### Psychological factors

The Hospital Anxiety and Depression Scale (HADS) was used to measure the degree of depression and anxiety symptoms over the past week (28). It is a 14-item scale, where seven items assess depression and seven items assess anxiety. The items are rated on a scale from 0 to 3, with higher scores indicating greater symptom severity. The cut-off points for the subscales are as follows: 0–7 for non-cases, 8–10 for doubtful cases, and 11–21 for clinical cases (28). The validity and reliability of both the English original and the Swedish version used in this study are well established (28, 29).

*The Pain Catastrophizing Scale (PCS)* measured pain-related catastrophizing within three subscales: helplessness, magnification, and rumination (30). The 13 items are scored on a 5-point scale (0 = not at all; 4 = all the time) and summed to produce a total score. Should read: Higher scores denote greater levels of catastrophizing, and a total score of 30 or above represents a clinically significant level of pain catastrophizing. The psychometric properties of the PCS are considered satisfactory (30).

### Predictor variables

The sociodemographic variables included age and education level.

*The Endometriosis Health Profile-30 (EHP-30)* was used to assess control and powerlessness, social support, and self-image. EHP-30 is an endometriosis-specific self-report measure used to assess health-related quality of life in individuals with endometriosis (31). It includes 30 core items divided into five core subscales measuring pain, control and powerlessness, self-image, emotional wellbeing, and social support. Additionally, there are six supplementary modules comprising 23 items divided into work, relationship with children, sexual relationship, feelings about the medical profession, feelings about treatment, and feelings about infertility. Each item is rated on a five-point scale: (0 = never; 4 = always). The scores in each domain are converted to scale of 0–100, where 0 indicates the best health status and 100 the worst. The psychometric properties of both the original and the Swedish version of the EHP-30 have been found to be satisfactory (31, 32).

### Analysis

LCA was used to identify subgroups among the participants. Based on a previous Swedish study of patients with chronic pain (12), five indicators were used in a finite mixture model: anxiety, depression, pain extent, pain intensity, and pain catastrophizing. To detect the optimal latent class solution, 2- to 6-class solutions were modelled. Enumeration indices for selecting the optimal solution included the adjusted Lo-Mendell-Rubin Likelihood Ratio Test (LMR-LRT) (33), Akaike information criteria (AIC), Bayesian information criteria (BIC), and sample-size-adjusted BIC (34). Specifically, we ran five models and collected the fit statistics listed above. The optimal model should show lower values on AIC, BIC, and sample size adjusted BIC, as well as higher entropy. The adjusted LMR-LRT produces a significant result (*p* < 0.05) if moving from k–1 to k latent classes leads to a statistically better model fit. Other than these fit statistics, we also considered a subgroup size smaller than 5% as problematic (34).

Following the identification of latent classes, five covariates (i.e., control and powerlessness, social support, self-image, age, and education level) were included as predictors of latent class membership. To extend the unconditional mixture model, a three-step approach (35) was employed using auxiliary variables, enabling the investigation of how latent class indicators exert their influence. Data were managed in R 4.4.0, and all major analyses were performed in Mplus 8.10 (36, 37).

## Results

### Participant characteristics

The study involved 113 female participants, with a mean age of 36.9 years (*SD* = 8.0; range = 21–61). The majority of participants (70.8%) were born in Sweden or another Nordic country. In terms of education, 62.5% had completed tertiary education, while 27.7% had completed secondary education. The mean pain extent among participants was 9.7 locations (SD = 6.7). Additionally, two participants (1.8%) reported only one pain location, while 96 participants (85.0%) had four or more pain locations. For pain intensity, 23.9% of participants reported mild pain (scores: 0–3), 28.4% reported moderate pain (scores: 4–6), and 47.7% reported severe pain (scores greater than 6). Notably, 54.9% had a clinically significant level of pain catastrophizing. Regarding mental health, results from the depression subscale showed that 47.8% were non-cases, 23.0% were doubtful cases, and 29.2% were clinical cases. The anxiety subscale revealed that 28.3% were non-cases, 25.7% were doubtful cases, and 46.0% were clinical cases. For more information, see Table 1.

**Table 1:** Participant characteristics (*N* = 113).

Variable	*n* (%) or M (SD)
Age (years)	36.9 (8.0)
Birthplace	
Sweden or Nordic Country	80 (70.8%)
Other country	33 (29.2%)
Education level	
Elementary school	8 (7.1%)
Upper secondary school	31 (27.7%)
University	70 (62.5%)
Other	3 (2.7%)
Pain extent (0–36)	9.7 (6.7)
Pain intensity	5.6 (2.7)
Pain catastrophizing	31.9 (12.3)
Depression	7.9 (4.4)
Anxiety	10.3 (4.7)

### Identifying latent classes

**Figure 1:**
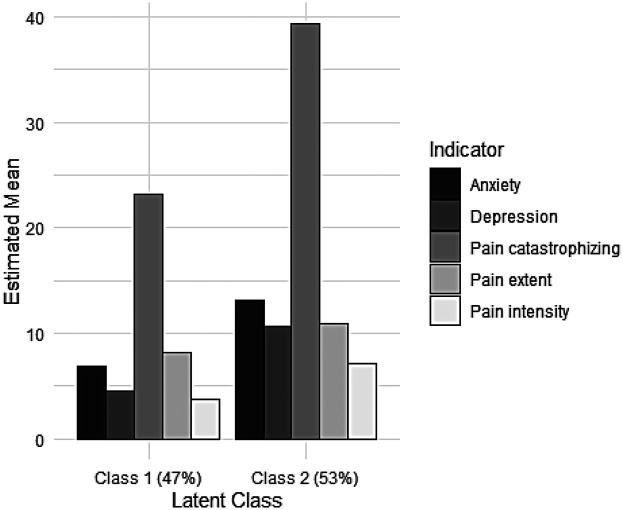
Estimated means of indicators in two latent classes. Class 1 means: Anxiety = 6.9; Depression = 4.6; Pain catastrophizing = 23.2; Pain extent = 8.2; Pain intensity = 3.8. Class 2 means: Anxiety = 13.2; Depression = 10.7; Pain catastrophizing = 39.3; Pain extent = 11.0; Pain intensity = 7.1.

To identify relevant clinical subgroups among the participants, LCA was used. As shown in Table 2, although key information criterion values (i.e., AIC, sample-size-adjusted BIC) decreased as the number of classes increased, their entropy metrics remained similar at approximately 0.80. Moreover, the 5- and 6-class solutions produced very small subgroups (i.e., classes with only four members), indicating these solutions were problematic. Judging from the adjusted LMR-LRT values, only the 2-class solution showed a statistical improvement from 1-class to 2-class, and further increases of class number did not enhance this fit. Thus, the 2-class solution was deemed optimal. Figure 1 presents the estimated means of these two latent classes (subgroups). Class 2 members clearly showed elevated levels across all indicators, suggesting they experienced greater psychological distress and more severe pain characteristics than Class 1 members.

**Table 2:** Summary of fit statistics of 2-to 6-class solutions.

Solution	AIC	BIC	SSABIC	Entropy	LMR-LRT (*p*)	Smallest subgroup size (%)
c2	3,383.55	3,427.19	3,376.62	0.81	131.69 (0.000)	53 (46.9%)
c3	3,369.59	3,429.59	3,360.06	0.82	25.08 (0.254)	27 (23.9%)
c4	3,360.21	3,436.58	3,348.08	0.82	20.65 (0.397)	19 (16.8%)
c5	3,348.86	3,441.59	3,334.14	0.83	19.43 (0.621)	4 (3.5%)
c6	3,341.69	3,450.79	3,324.37	0.83	16.68 (0.770)	4 (3.5%)

Note: AIC, Akaike information criteria; BIC, Bayesian information criteria; SSABIC, sample-size- adjusted Bayesian information criteria; LMR-LRT, adjusted Lo-Mendell-Rubin Likelihood Ratio Test. *p*: *p*-value.

### Covariates of latent class membership

Logistic regression using the three-step approach identified two covariates with statistical significance (Table 3). Compared with Class 1, Class 2 members reported worse control and powerlessness (*b* = 0.29, *p* = .013, *OR* = 1.34) and greater deficits in perceived social support (*b* = 0.36, *p* = .001, *OR* = 1.44).

**Table 3:** Logistic regression predicting latent class membership.

Variable	Estimate	S.E.	*P*-value	Odds ratio
Control and powerlessness	0.29	0.12	0.013	1.34
Social support	0.36	0.11	0.001	1.44
Self image	0.01	0.13	0.948	1.01
Age	0.04	0.06	0.506	1.04
Education	1.21	0.83	0.145	3.36

Note: Class 1 was used as the reference group. Control and powerlessness, social support, and self image were measured with the Endometriosis Health Profile-30.

## Discussion

This study aimed to use clinical factors from the biopsychosocial model (including pain intensity, pain extent, depression, anxiety, and pain catastrophizing) to identify distinct subgroups of individuals with endometriosis, receiving care at a tertiary clinic. Two distinct subgroups were discerned: Class 2, characterized by a high biopsychosocial (BPS) burden with elevated psychological distress and severe pain, and Class 1, defined by a lower BPS burden with comparatively mild symptoms across domains.

These subgroups were also compared in terms of sociodemographic characteristics and different indices of functioning. Notably, the high-BPS burden group reported worse control/powerlessness and greater deficits in perceived social support. To our knowledge, this is the first study to cluster clinically verified endometriosis patients based on the biopsychosocial model, which considers the dynamic interaction between biological, psychological, and social factors (19, 22). Overall, the findings extend the chronic pain literature into the field of gynaecological care, highlighting the potential benefits of interdisciplinary integration for improving clinical understanding and treatment.

The high-BPS burden group represented 53% of the sample, indicating that a substantial proportion of patients at a tertiary care center experience complex, multifaceted difficulties. This underscores the need for comprehensive assessments incorporating core components of the biopsychosocial model when evaluating individuals with endometriosis (22). The findings also emphasize the need to move toward individualized, stratified, phenotype-based treatment approaches in endometriosis care, tailoring interventions based on biopsychosocial burden rather than relying on one-size-fits-all strategies.

Overall, psychological strain was high across the entire sample, with 54.9% reporting a clinically significant level of pain catastrophizing, 46.0% meeting criteria as clinical cases of anxiety, and 29.2% as clinical cases of depression. These findings are consistent with existing research highlighting elevated rates of psychiatric comorbidities and pain catastrophizing among individuals with endometriosis (8, 9, 11, 38). Pain catastrophizing, in particular, has been shown to moderate the relationship between pain severity and symptoms of depression in individuals with endometriosis, and to have strong associations with quality of life (39, 40). Depression, anxiety, and catastrophizing are also considered key factors in pain-related functioning (41, 42). In line with this, Class 2 participants exceeded clinical thresholds for all three indicators and exhibited more severe pain profiles. The group also reported feeling worse powerlessness, an experience conceptually linked to helplessness, further emphasizing the role of catastrophizing in shaping clinical presentation.

The elevated levels of depression and anxiety reaching clinical significance in the high-BPS burden group align with a growing body of evidence in chronic pain and pain phenotype research, which identifies subgroups characterized by heightened pain severity and pronounced emotional symptoms (23, 43). A positive correlation between pain intensity and anxiety symptoms has been demonstrated in patients with endometriosis (44). Moreover, anxiety, which may be triggered by specific gynecological procedures, is known to amplify the perception of pain (45). The observed overlap between pain and emotional symptoms in Class 2 suggests a bidirectional relationship, emphasizing the central role of mental health in the clinical manifestation of pain (43, 46). These findings further underscore the contribution of anxiety and depression to more severe clinical presentations in individuals with endometriosis. The additive effect of emotional symptoms in the high-BPS burden group potentially requires targeted interventions addressing these symptoms specifically. Hence, psychological therapies such as Cognitive Behavioral Therapy (CBT), with a sound evidence base in chronic pain, may be of particular importance in this subgroup of individuals with endometriosis. This aligns with chronic pain management guidelines where such interventions are the front-line treatment, but are not yet routine in endometriosis care (47).

Regarding pain characteristics, the average number of pain locations was 9.7 (on a scale from 0 to 36), suggesting that most participants experienced regional pain, as pain is typically described as localized, regional, or widespread (48). Only 1.8% reported having only one pain location. A total of 85.0% reported having four or more pain locations, potentially indicating pain spreading. For pain intensity 28.4% reported moderate pain, and 47.7% reported severe pain.

In the high-BPS burden group, severe pain intensity (M = 7.1) was observed, compared to moderate pain (M = 3.8) in the low-BPS burden group, along with more pronounced spreading of pain (11.0 locations compared to 8.2 in the low-BPS burden group). Prior studies have identified pain extent as a clinically meaningful marker (12). More widespread pain has also been associated with a longer pain duration and a more severe clinical picture with strong associations to health and pain aspects (pain intensity and pain interference) (49, 50). In addition to indicating severity, pain spreading can also, to some extent, predict poorer outcomes of pain rehabilitation programs (50). Pain spreading is also linked to central sensitization and nociplastic pain mechanisms, making the slightly higher number of pain locations in the high-BPS group clinically noteworthy (51). However, it should be noted that 8 or 11 locations indicate mild to moderate pain spreading (50). Nonetheless, even this relatively subtle difference in pain spreading between the high- and low-BPS burden groups may have clinical implications and should be considered in clinical evaluations.

Pain intensity, by contrast, differed more distinctly with moderate pain ratings in the low-BPS burden group and severe pain ratings in high-BPS burden group. This indicates that high pain intensity is coupled to a more severe clinical picture, which is not surprising. The result aligns with previous studies showing that endometriosis and high pain intensity are correlated with lower quality of life (39, 52). The findings are also in line with previous research demonstrating that individuals with higher pain intensity report elevated pain-related disability and emotional distress (52, 53).

Clinical assessment of patients with endometriosis may benefit from integrating core principles from the biopsychosocial model. Treatment strategies for endometriosis have traditionally been biomedical, yet they often yield suboptimal outcomes for a substantial subgroup of patients. The characteristics of Class 2 reveal significant, complex difficulties in several areas of the biopsychosocial model, potentially pointing to a need for intensive, multidisciplinary interventions grounded in the biopsychosocial model at a tertiary level of care. The low-BPS burden group (i.e., Class 1) may benefit from less resource-intensive, monodisciplinary interventions. Implementing subgroup-based treatment strategies could significantly improve quality of life for individuals with endometriosis. Stratifying patients based on their biopsychosocial profile may also enhance allocation of healthcare resources, providing multimodal care to those with higher psychosocial burden, and primarily unimodal care to those with lower burden. Further longitudinal studies are needed to validate these findings and guide the development of tailored treatment approaches.

Our study had several limitations. This exploratory study utilized real-life clinical data from eligible patients referred to the Endometriosis Clinic, Skåne University Hospital during the study period. As a result, a formal sample size calculation was not performed prior to data collection. While this approach reflects routine clinical practice and enhances the study's ecological validity, it may limit the statistical power and generalizability of the findings. Future research with predefined sample size estimations is needed to confirm and expand upon these results. The cross-sectional nature of this study also hindered us from analyzing causality. Future studies should investigate how biopsychosocial profiles evolve over time and how they respond to targeted interventions. Diagnostic confirmation through laparoscopy or ultrasound enhances the study by ensuring diagnostic accuracy and clinical consistency across the cohort. However, this approach may limit generalizability, as it excludes important subgroups of individuals with endometriosis, such as those who are asymptomatic or remain undiagnosed. Additionally, this study relied on self-report for other data which may introduce bias. The sample was relatively small and taken from a single-center tertiary clinic based within the Swedish national health services, serving as a regional, specialist center providing healthcare services for individuals with endometriosis, where a small subgroup had planned surgical interventions. Hence, the high proportion of approximately 50% with a higher degree of biopsychosocial symptoms might not generalize to other levels of care, as the sample most likely represents patients with a higher disease burden. Future research should aim to replicate these findings in both primary and secondary care settings, as well as in more diverse populations, including asymptomatic individuals and those identified through community-based screening. Broadening the research scope in this way will help improve generalizability and better reflect the full spectrum of individuals affected by endometriosis. Additionally, while this study focused on biological and psychological indicators, social dimensions were underrepresented. Future research should incorporate explicit social variables—such as employment status, financial strain, or relationship functioning—to more fully capture biopsychosocial dynamics. Integration of biopsychosocial phenotypes with biological markers or disease stage could also help develop a more comprehensive classification system.

In conclusion, this study identified two distinct biopsychosocial subgroups among individuals with endometriosis, highlighting the relevance of psychological and pain-related factors in clinical presentation. The high-BPS burden group showed significant emotional distress and severe pain characteristics, suggesting the need for multidisciplinary care. Integrating biopsychosocial assessment into routine practice may support more personalized and effective treatment strategies, improving outcomes and resource allocation in endometriosis care.

## Data Availability

The datasets presented in this article are not readily available due to reasons of sensitivity. Requests to access the datasets should be directed to sophia.akerblom@med.lu.se.
